# Preservation of Preloaded DMEK Lenticules in Dextran and Non-Dextran-Based Organ Culture Medium

**DOI:** 10.1155/2016/5830835

**Published:** 2016-11-22

**Authors:** Mohit Parekh, Alessandro Ruzza, Stefano Ferrari, Diego Ponzin

**Affiliations:** International Center for Ocular Physiopathology, Fondazione Banca degli Occhi del Veneto Onlus, Venice, Italy

## Abstract

*Purpose.* To determine the optimum preservation conditions for preloading DMEK lenticules using organ culture system.* Methods.* 8.5 mm DMEK lenticules were stripped and preserved with endothelium flap-in for 4 days at RT in an IOL cartridge that was blocked with rubber stoppers from each end. In C1, tissues were collected from tissue culture medium (TCM) and preserved in TCM. In C2, tissues were collected from transport medium (TCM + 6% dextran T500) (TM) and preserved in TM. In C3, tissues were collected from TCM and preserved in TM. Mortality, glucose uptake, histological staining, tight junctions and cell apoptosis were studied post-preservation.* Results.* Mortality in C1, C2, and C3 were 49.40%, 8.53%, and 27.74%, with 40.7%, 13%, and 41.8% uncovered areas. Glucose uptake (mg/mL) was 0.32, 0.43, and 0.56 in C1, C2, and C3. PAS staining showed presence of DM and endothelium in C2 but not in C1 and with fewer cells in C3. ZO-1 was expressed in all the conditions. Polymorphism was higher in C1 and C3. Mild apoptosis was observed in C3.* Conclusions.* Dextran may play an important role in preserving the endothelial cells before and after stripping for trifolded (endothelium-in) preloaded DMEK lenticules.

## 1. Introduction

Descemet's Membrane Endothelial Keratoplasty (DMEK) is a type of corneal surgery, which allows the transplantation of Descemet's Membrane (DM) and endothelium [1–4]. DMEK has its own advantages as compared to penetrating keratoplasty (PK) in terms of better optical quality, early visual rehabilitation, and less postoperative astigmatism with a much protected eye. As it does not involve excision of the entire cornea (optic zone) from the patient's eye like PK, it is considered a safer surgery. Various techniques have been identified for the preparation of this highly fragile tissue [5–12].

We at the Veneto Eye Bank Foundation have recently started providing preloaded tissues for DSAEK and UT-DSAEK surgeries, a step further to precut tissues [13, 14]. This reduces the time and efforts in surgical theatre, increases efficiency of the DSAEK surgery, and allows validated tissue to be used. Eye bank prepared DMEK tissues are usually prestripped, rolled, or prebubbled and shipped to the operating room [8, 12]. In our institute, these tissues are stripped and currently preserved in transport medium [TM] (tissue culture medium + 6% dextran T500) which is a deswelling medium required for transportation. As the tissue is only comprised of DM and endothelium, the requirement of dextran is not justified for preserving DMEK lenticule. However, due to the properties of dextran, which may be useful for keeping the cells adherent to the extracellular matrix, its evaluation therefore becomes necessary.

Tissue culture medium (TCM) is the most commonly used corneal storage media in Europe while hypothermic-based preservation method is pursued in America and most of the world. As the tissue preservation is important to keep the endothelium viable, it becomes necessary to investigate the optimum condition to preload a DMEK lenticule, which is the next advancement in the field of endothelial keratoplasty [15]. Preloading is likely to reduce the undesired effects that are seen while shipping the tissues as free floating or prestripped and allows transplanting a validated tissue. Thus, the aim of this paper is to study the optimum preservation conditions (medium with and without dextran) and to evaluate the possibility of preserving the DMEK lenticules flapped (trifolded) in a closed chamber, that is, to preload and provide a ready-to-use tissue to the surgeons for transplantation with minimal manipulations.

## 2. Materials and Methods

### 2.1. Ethical Statement

Thirty human donor corneal tissues were collected from the Veneto Eye Bank Foundation, (Venice, Italy) with a written consent from the donor's next of kin to be used for research.

### 2.2. Media Constituents

TCM was composed of 2% newborn calf serum with MEM-Earle as a base medium along with 25 mM Hepes buffer, 26 mM sodium bicarbonate, 1 mM pyruvate, 2 mM glutamine, 250 ng/mL amphotericin B, 100 IU/mL penicillin G, and 100 mg/mL streptomycin. TM was composed of TCM incorporated with 6% dextran T500. TCM and TM were prepared in house (FBOV, Mestre, Italy) with full regulatory compliance.

### 2.3. Preevaluation

All the corneas were preserved in TCM before the study. However, to load the tissues and study the effect of the preservation medium on the tissues, ten corneas were further preserved in TM. The endothelial cells were evaluated using a hypotonic sucrose solution and the viability was checked using trypan blue staining for 30 seconds followed by washing the cells with phosphate-buffered saline (PBS). Corneal thickness from the TCM and TM groups was recorded before peeling using Optical Coherence Tomography (OCT SS-1000, Tomey, Nagoya, Japan).

### 2.4. Preservation Conditions

The tissues were collected and preserved in different conditions, as described in Table 1.

### 2.5. Stripping and Loading

The procedure was carried out as described in our previously published article [15]. In brief, the corneas [*n* = 30] were mounted on a vacuum punch base and secured. The corneas were gently tapped on the endothelial side and a 9.5 mm superficial cut was created using a Moria punch (Moria, Antony, France). The endothelium was stained using trypan blue for 20 seconds to determine the cut area. After removing the periphery, the central DMEK lenticule was excised and placed back on the tissue with endothelium facing the air. The prestripped membrane was punched again to excise an 8.5 mm (Moria, Antony, France) lenticule. The peripheral remnants were removed. The lenticule was folded (trifold) with endothelium, in position using an acute forceps. The lenticule was then gently moved in the preservation chamber of a 2.2 intraocular lens (IOL) cartridge (Viscoject, Wolfhalden, Switzerland) and was further pulled inside the funnel of the cartridge using 25G microincision forceps from the funnel end. The funnel was filled with the preservation medium as listed in Table 1 before the lenticule was inserted and was later filled completely. The funnel and the back entrance were closed using rubber stoppers and the entire system was preserved in the media as listed in Table 1. The lenticules were preserved for 4 days at room temperature. The stopper was removed and the tissues were released out from the funnel pore using the microincision forceps and analyzed as described below.

### 2.6. Endothelial Cell Evaluation

All the lenticules [*n* = 30] were stained after storage using trypan blue for 20 seconds and washed with phosphate-buffered saline (PBS). The lenticules were exposed to sucrose solution in a petri plate. The endothelial cell density (ECD) and mortality were counted using a 10 × 10 mm reticule (grid) inserted in the eyepiece of an inverted microscope (Primovert; Zeiss, Milan, Italy) at 100x magnification by masked observers.

### 2.7. Glucose Uptake of the Preserved Lenticules to Determine the Metabolic Activity

Glucose uptake was determined from the preservation media of all the samples [*n* = 30] after 4 days of preservation (poststorage) in an IOL cartridge. This helped to check the metabolic activity of the endothelial cells when preserved* in vitro*. Quantitative analysis was performed using D-Glucose HK kit (Megazyme International Ireland Ltd, Bray Business Park, Bray, Co., Wicklow, Ireland) after preservation.

### 2.8. Histological Analysis to Determine the Presence of Endothelial Cells on DM, Collagen Fibrils, or Stromal Residues

The lenticules were opened up after storage before processing them for histological analysis. The presence of Descemet's Membrane, collagen fibers, and endothelium was investigated [*n* = 9; *n* = 3 from each condition]. The tissue was fixed in 4% PFA overnight followed by washing it with sucrose solution at 7.5%, 15%, and 30% for 15 minutes each. Final washing was carried out with PBS and the tissues were embedded in Optimal Cutting Temperature (OCT) for microtome cutting. Periodic Acid-Schiff (PAS) staining was performed on all the samples and sections were viewed at 10x magnifications in order to check the variability and reproducibility in tissue selection and preservation performances along with stromal interference, if any.

### 2.9. Antibody Staining to Determine the Polymorphism, Expression of Tight Junctional Proteins, and Cell Apoptosis Study, for Cellular Integrity after Preservation

#### 2.9.1. Tissue Fixation and Preparation for Cell Apoptosis and Immunostaining

The tissues (*n* = 21; *n* = 7 from each condition) were opened up after storage before checking cell apoptosis and immunostaining. The preserved tissues were fixed in 4% paraformaldehyde (PFA) at 4°C overnight.

#### 2.9.2. Immunostaining with Zonula Occludens-1 (ZO-1)

Twelve tissues [*n* = 4 from each condition], previously treated as described above, were permeabilized with 0.5% Triton X-500 in PBS for 30 minutes. After blocking with 2% goat serum, the tissues were incubated overnight at 4°C with a primary antibody (Zonula Occludens-1 [ZO-1], 1 : 500 dilution). The samples were incubated with goat anti-mouse fluorescein isothiocyanate- (FITC-) conjugated secondary antibody in 20% goat serum for 3 hours at room temperature. Mounting medium containing 4,6-diamidino-2-phenylindole (DAPI) was used to stain the nuclei. After each step, the cells were washed 3 times with 10x PBS. Cells were examined with an LSM 510 Meta Laser Scanning Microscope (Zeiss, Milan, Italy). Examination was performed under the ultraviolet light or by excitation at 488 nm or 547 nm, and subsequent detection of the fluorescence was obtained.

#### 2.9.3. Cell Apoptosis Using Terminal Deoxynucleotidyl Transferase Deoxyuridine Triphosphate Nick-End Labeling Assay

Cell apoptosis was performed as described in the manufacturer's protocol for TACS 2 terminal deoxynucleotidyl transferase (TdT) diaminobenzidine (DAB)* in situ* apoptosis detection kit (Cat# 4810-30-K; Trevigen, Maryland, USA). One separate positive sample was induced with apoptosis using TACS nuclease and nine samples [*n* = 3 from each of the Cs] were viewed at 100x magnifications of an inverted microscope. The images were analyzed using ZEN (Zeiss, Milan, Italy) software.

### 2.10. Statistical Analysis

Student's *t*-test was employed to check statistical significance between the different groups. *p* < 0.05 was deemed statistically significant.

## 3. Results

### 3.1. Donor Characteristics and Preevaluation [*n* =* 30*]

The average age of the donor was 67.1 (±6.20) years with male : female donor ratio of 24 : 6. Average postmortem time was 14.6 (±6.45) hours. All the corneas were previously preserved in TCM with an average preservation time of 14.27 (±6.09) days. The tissues were further preserved in TM for 3.4 (±2.72) days before stripping. The average endothelial cell density recorded was 2203.33 (±335.52) cells/mm^2^ with initial mortality of 0.2 (±0.54)%. Successful peeling was observed in 76% of cases when the tissues were collected from TCM whereas 100% tissues were peeled successfully when the tissues were collected from TM. Average stripping time for C1 and C3 was 25 minutes and that for C2 was 19 minutes. Average loading time was 5 minutes for all the tissues. The tissues collected from TCM had a higher thickness of  963.5 (±77.02) *μ*m as compared to TM, which was 570.3 (±49.47) *μ*m thick before stripping. The tissues from TM showed thickness similar to* in vivo* corneas as they were deswelled.

### 3.2. Lower Endothelial Cell Loss in Dextran-Based Media [*n* =* 30*]

**Figure 1:**
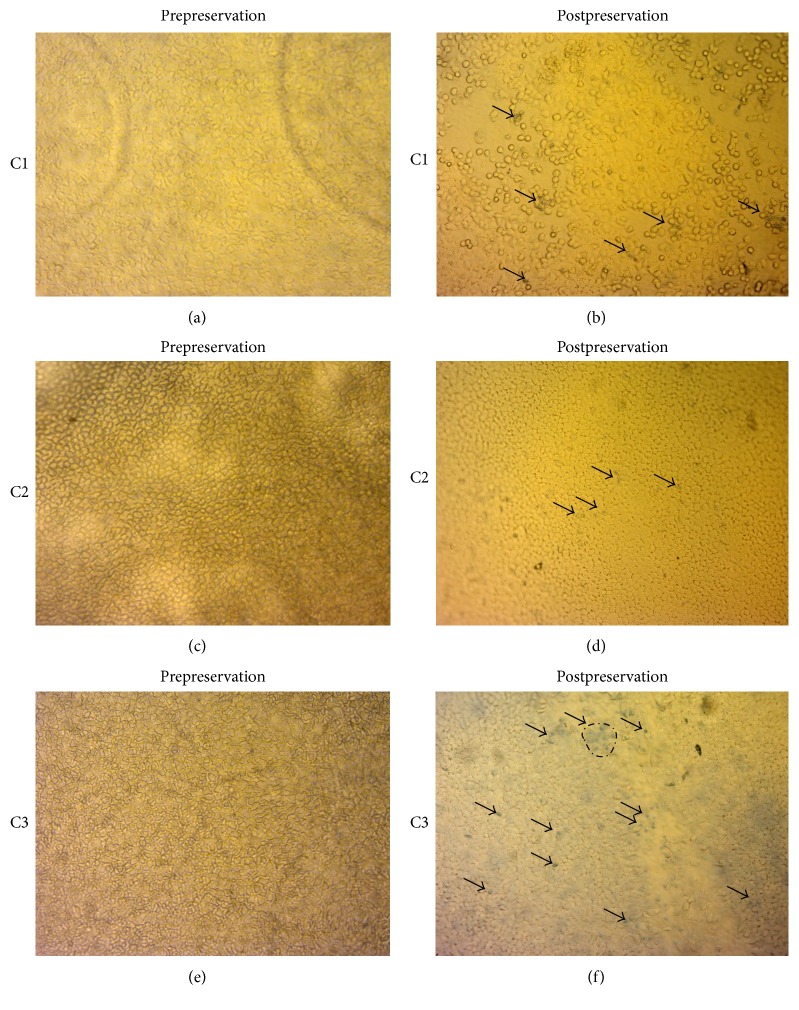
Endothelial cell density, mortality, and uncovered areas of preloaded DMEK lenticules at 100 magnification. (a, c, e) show healthy endothelium that was found after stripping with minimal mortality in different conditions as listed. (b, d, f) show mortality and uncovered areas in each condition. C1 and C3 showed high mortality (trypan blue positive cells marked with arrow) and uncovered areas as compared to C2, which showed minimal mortality.

ECD (cells/mm^2^) after preservation in C1, C2, and C3 was found to be 1130 (±944.05), 1950 (±108.01), and 1970 (±512.18), and the mortality (%) was 25.94 (±44.23), 3.8 (±7.74), and 21.8 (±36.41), respectively. It was also observed that the cells detached from the lenticule during preservation and therefore the uncovered areas (%) determined for C1, C2, and C3 were 40.7 (±47.96), 13.0 (±18.55), and 41.8 (±37.77). Loading to postpreservation in C1 showed statistically significant endothelial cell loss (*p* = 0.0051); however, C2 (*p* = 0.1092) and C3 (*p* = 0.0819) did not show statistical significance, although the average ECL observed in C3 was high.  Figure 1 shows how the lenticules obtained from corneas preserved in C1, C2, and C3, respectively, appear after stripping (Figures 1(a), 1(c), and 1(e)) and after preservation (Figures 1(b), 1(d), and 1(f)).

### 3.3. Glucose Was Utilized in All the Media [*n* =* 30*]

Average glucose uptake by the endothelial cells in the preservation chamber for 4 days at room temperature was 0.32 (±0.18) mg/mL from C1, 0.43 (±0.27) mg/mL from C2, and 0.56 (±0.18) mg/mL from C3, which is one-third to half of the total amount of glucose present in the media. These results are similar to those showed in the preloaded DSAEK lenticules [13]. However, there was no statistical difference seen between either group (*p* > 0.05).

### 3.4. Presence of DM and Endothelial Sheet on the Excised DMEK Tissues from Dextran-Based Media [*n* =* 9*]

**Figure 2:**
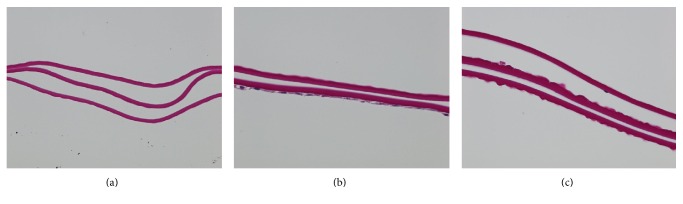
Histological analysis on preloaded DMEK lenticules after preservation at 100x magnification. (a) C1 showed no presence of endothelium due to major cell fall-off, (b) C2 showed presence of endothelium and DM without any stromal remnants, and (c) C3 showed limited endothelium.

The analyzed tissues (100x magnification using PAS staining) showed no presence of endothelial cells, but only the DM (Figure 2(a)) in C1. This may be a reason due to high “fall-off” rate of the cells from C1. As observed in Figure 1, the cells do not maintain a hexagonal shape and have turned themselves into circular shape (stressed) along with high mortality as seen using trypan blue staining. Descemet's Membrane and endothelium (found in one of the tissues) without any collagen fibrils or attached stromal residues in C2 are seen in Figure 2(b). However, lesser endothelial cells were found in lenticules from C3 (Figure 2(c)). This phenomenon can be due to the presence of dextran in the preservation medium after loading in the cartridge. Preservation of endothelial cells with DM was found in C2.

### 3.5. Expression of Tight Junctional Proteins and Polymorphism on the Endothelial Cells after Preservation [*n* =* 12*]

**Figure 3:**
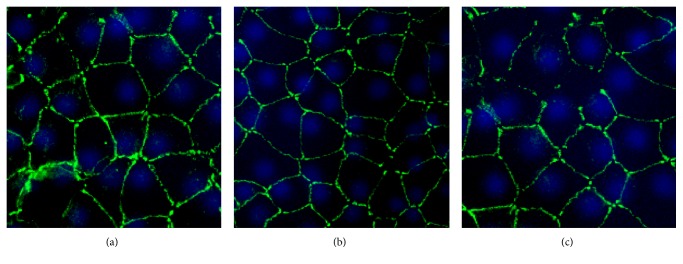
Immunostaining results using ZO-1 marker for expression of tight junctional proteins in the preloaded DMEK lenticules visualized at 400x magnification. Preserved cells showed expression of these proteins confirming the integrity of the endothelial cells. However, ((a) and (c)) C1 and C3, respectively, showed some areas without endothelial cell borders and hence loss of tight junctions whereas (b) C2 expressed tight junction protein with less polymorphism compared to C1 and C3. Hexagonality of the cells was observed in C2.

The endothelial cells showed expression of tight junction protein (ZO-1) after preservation as seen in Figure 3 at 400x magnification. C1 and C3 (Figures 3(a) and 3(c), resp.) showed loss of hexagonality; most of the cells were polygonal to circular. Tight junctional protein was not consistent in C3 but was intense in C1 at many areas. C2 (Figure 3(b)) showed endothelial cell mosaic with more hexagonal shaped cells and the conservation of tight junctional proteins in the preloaded DMEK lenticules.

### 3.6. No Cell Apoptosis from Dextran-Based Media [*n* =* 9*]

**Figure 4:**
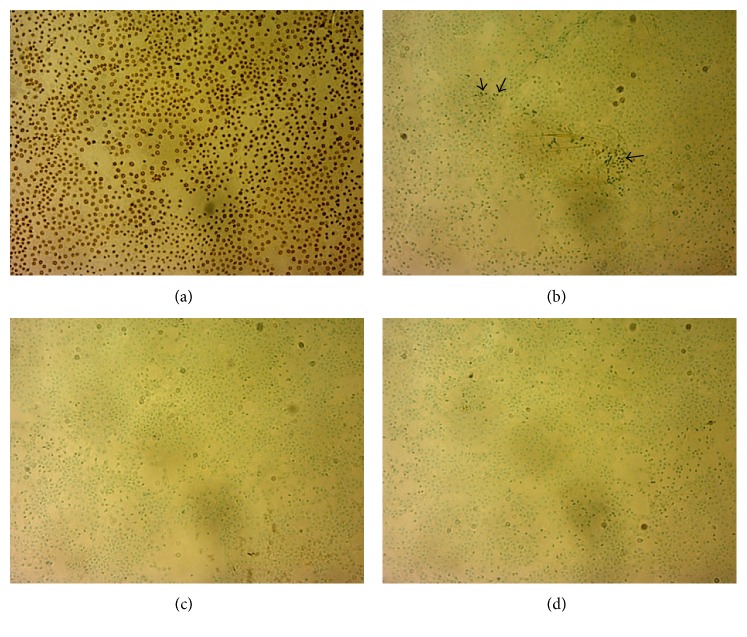
Cell apoptosis after preservation of preloaded DMEK lenticules. (a) Induced apoptosis on the control sample at 100x magnification. (b) C1 showed mild apoptosis whereas ((c) and (d)) C2 and C3 did not show any apoptosis after preservation and the nucleus was visible with methyl green counterstain at 100x magnification. Hence it was determined that the preservation of the DMEK lenticule in a cartridge for 4 days does not have any drastic damage on the endothelial cells.

Control cells were induced with apoptosis using TACS nuclease (Figure 4(a)) to compare them with the samples. C1 showed apoptosis after preservation at 100x magnification (Figure 4(b)). However, C2 and C3 did not show any apoptosis after preservation (Figures 4(c) and 4(d)). C2 and C3 were preserved in dextran-based medium and we assume that may have prevented the cells from apoptosis.

## 4. Discussion

As indicated in Introduction, DMEK requires high surgical skills, but there are ways to ameliorate the quality of the tissues distributed to surgeons and help them perform an easier transplantation. One of the methods is to check whether the preservation technique may affect the quality of the tissue. Dextran has been widely used to deswell the cornea after the tissue has been swollen during the TCM preservation phase. The number of viable endothelial cells and a thickness that is similar to the* in vivo* conditions are important for a successful corneal transplantation.

Organ culture offers several advantages in terms of sterility checks, longer duration of preservation, and hence a proper planning of the surgery [16]. Although storage in dextran-containing medium is not obligatory for preparing the tissues for DMEK surgery, the important parameters like endothelial cell density and smoothness of the DM should be considered before the surgery. It has been suggested that, with higher number of functional cells, the dislocation chances of DMEK decrease and the adherence increases [16]. To our knowledge, there has not been any study in the literature that shows the comparison of different storage media for preloaded DMEK preservation in a closed chamber like an IOL cartridge.

Previously, it has been shown that the preservation medium has no effect on the rate of successful peeling of DMEK tissues [16]. However, we observed that when the tissues are thick (stromal thickness), that is, approximately over 900 *μ*m, the success rate changes as compared to the normal thickness of the graft, which has regained its original thickness (approximately 550 *μ*m). We observed 76% successful peeling cases with higher thickness where the tissues were preserved in TCM as compared to 100% successful cases when the tissues were preserved in TM. Peripheral tearing that resulted in the loss of tissues eventually was the major reason when the tissues were excised from TCM. The stroma was thick and the punch for creating a superficial cut does not direct a regular cut in an uneven stromal base when the tissues were taken from TCM. On the contrary, when the tissues were obtained from TM, due to the regular thickness and a more constant cornea throughout, the cut obtained was precise and the excision was accurate without any peripheral tears. Therefore, to standardize the peeling technique, the tissues can be obtained from TM or from dextran-based media for peeling.

Further, the tissues are usually prebubbled, prestripped, or prerolled and preserved in the respective media [8, 12]. However, there has not been any study that reports about a completely excised and preloaded tissue. Therefore, we tried to preload the tissue in a closed chamber (the IOL cartridge). Tissues that were preloaded did not show any damage in terms of cellular morphology or molecular integrity. The glucose uptake study showed similar results to those obtained with preloaded DSAEK grafts, thus concluding that active metabolism and functional activities of the endothelium continue for at least 4 days at RT [13]. Histology confirmed the presence of DM and endothelium without any stromal residues in all the cases but the presence of endothelium was definite when the tissues were excised from C2. The cellular apoptosis was observed in C1; it was not due to the preloading but due to the preservation medium (TCM). Cell apoptosis has been reported to incur in the tissues that are preserved in TCM for a long time [17]. Therefore, dextran seems to play an important role in maintaining the functionality of the cells, while preloading the DMEK grafts.

Endothelium, if flapped in, does not have statistically more damage compared to endothelium when flapped outward (which is the natural phenomenon) [18]. However, we recommend that the tissues should be flapped in (a) to avoid any possible friction between the endothelial cells and the cartridge during preservation, transportation, and transplantation and (b) to reduce the time of opening the graft inside the recipient eye [18]. The average endothelial cell loss (ECL) [%] (average of mortality and uncovered areas) after preservation of the preloaded DMEK lenticule was found to be 45.05 (±46.61), 10.77 (±15.99), and 34.77 (±37.37) in C1, C2, and C3, respectively. The endothelial cell mortality cannot be considered high because of the following: (1) the tissue is punched again at the eye bank after stripping and is folded and loaded in the cartridge where there is some endothelial cell loss observed; this step is currently carried out in the surgical theatre where the surgeons do not check the endothelial cell loss after this step; (2) the tissue is then preserved in closed chamber (IOL cartridge) in the same orientation without any free movement and some cells may be damaged due to friction; (3) the tissues obtained for this research are not suitable for transplantation because of low endothelial cell count and previous mortality; therefore it may have some additional mortality due to the health of the tissue in general. The results can still be extrapolated to the transplantable grade tissues as they have high endothelial cell density (between 1800 and 2000 cells/mm^2^) for research. As per our previously reported article, there is a learning curve to preload a DMEK graft and after the learning curve the average endothelial cell mortality and uncovered areas after preservation in dextran-based media were 3.55% (±5.79%) and 7.8% (±14.13%), respectively. Overall endothelial cell loss (ECL) after preservation in dextran-based medium was 4.35%, which is less than the endothelial cell loss observed here that is due to the learning curve [15]. Moreover, dextran is negatively charged and therefore the media repel the endothelial cells allowing a higher cell adherence to the extracellular matrix, the DM. This property of dextran may also help to reduce the cell fall-off and highlight that the tissue should be preserved in dextran-based medium. The tissues from TCM have two major disadvantages such as (a) lower successful peeling rate and (b) higher endothelial cell loss. We have also noticed that the tissues preserved in C2 have a better flexibility, lower stiffness, and high cell to extracellular matrix adherence (data under consideration for publication) as compared to the other conditions.

In conclusion, preloading a DMEK tissue may facilitate DMEK to be more successful in terms of transplanting a validated graft along with a comparatively short surgical procedure, low costs, and logistic requirements. Dextran may therefore have an important role during preservation, preparation, and transportation, and hence it should be considered in the medium before prestripping or preloading a DMEK lenticule.

## Figures and Tables

**Table 1:** Different conditions of preservation media to store preloaded DMEK lenticules.

	C1	C2	C3
Tissues collected from	TCM	TM	TCM
	Stripping	Stripping	Stripping
Preserved in	TCM	TM	TM
	Analysis	Analysis	Analysis

C1, C2, and C3: condition 1, condition 2, and condition 3.

TCM: tissue culture medium.

TM: transport medium.
